# Ratio of diastolic to systolic blood pressure represents renal resistive index

**DOI:** 10.1038/s41371-019-0264-1

**Published:** 2019-10-04

**Authors:** Tetsuya Akaishi, Michiaki Abe, Takashi Miki, Mika Miki, Yasuharu Funamizu, Sadayoshi Ito, Takaaki Abe, Tadashi Ishii

**Affiliations:** 1 Department of Education and Support for Regional Medicine, Tohoku University Hospital, Sendai, Japan; 2 Department of Nephrology, Endocrinology and Vascular Medicine, Tohoku University, Sendai, Japan; 3 Clinical Physiology Center, Tohoku University Hospital, Sendai, Japan

**Keywords:** Hypertension, Chronic kidney disease

## Abstract

Increased intrarenal vascular resistance is suggested to accompany chronic kidney diseases (CKD), which is known to be closely associated with hypertension. However, there are few studies that have examined the relationship between blood pressure and intrarenal vascular resistance. Renal color Doppler ultrasonography is one method that can non-invasively evaluate intrarenal vascular resistance. In this study, we comprehensively studied the correlations between ultrasonic parameters and blood pressure indices to elucidate their relationships. In total, 162 patients with suspected CKD were enrolled for this study. Demographics, blood pressure, blood test, urine test, and renal color Doppler ultrasonography data were obtained. The ratio of diastolic to systolic blood pressure (D/S ratio) and pulse pressure were calculated. Our results indicated strong negative correlations between the renal resistive index (RI) values in all four of the studied kidney regions and the D/S ratio. The RI values also showed significant correlations with diastolic pressure and pulse pressure, but they were weaker. Partial correlation coefficients between pulse pressure, mean arterial pressure, D/S ratio, and RI showed that D/S ratio significantly correlated with RI, but pulse pressure or mean arterial pressure did not. Systolic blood pressure did not correlate with any of the studied ultrasonic values. The negative correlation between RI values and the D/S ratio was still observed in subjects without renal dysfunction or any medications. In conclusion, D/S ratio, rather than pulse pressure or mean arterial pressure, would be the most appropriate index to estimate/calculate/judge intrarenal vascular resistance.

## Introduction

Renal color Doppler ultrasonography is a useful noninvasive physiological examination for the assessment and follow-up of patients with chronic kidney diseases (CKD) [1–4]. In addition to kidney size, routine ultrasonic indices—end-diastolic velocity (EDV), peak systolic velocity (PSV), and resistive index (RI)—are usually measured in multiple regions from the central to peripheral kidney [3, 5]. Because renal color Doppler ultrasonography was introduced only in the past few decades, its clinical usefulness and guidelines for interpretation are still being determined. In general, long-term blood pressure management of CKD patients is important [6–8]. Decline in renal function is closely associated with elevated blood pressure [9]. Although the connections between hypertension and CKD have been widely studied, the relationship between findings of renal color Doppler ultrasonography and blood pressure is unclear. If such an association is established, the routine measurement of daily blood pressure would certainly offer much more clinical information and also enable more correct interpretations of measured blood pressure, which could result in better medical approaches and interventions for each CKD patient.

Pulse pressure (PP) that is known to reflect the arterial stiffness or mean arterial pressure has been shown to correlate with renal vascular resistance [10, 11], but a simple ratio of diastolic to systolic blood pressure (SBP) has never been evaluated before. In this study, to elucidate the association between measured blood pressure and intrarenal hemodynamics, we simultaneously acquired blood pressure and renal color Doppler ultrasonography data from suspected CKD patients with or without renal dysfunction and examined the correlations between these measurements.

## Methods

### Subject enrolment

We collected clinical and blood test data from all subjects who were examined using renal color Doppler ultrasonography between 2007 and 2017 in our facility for suspected CKD. MA, TM, MM, and YF performed the test during this period. In total, 204 subjects with or without CKD were evaluated using their clinical and blood tests as well as renal color Doppler ultrasonography. From this population, a total of 42 patients with apparent renal arterial stenosis, solitary kidney, or under treatment with artificial dialysis were excluded, resulting in a total of 162 CKD patients for this study.

### Collected clinical data and measured variables

The demographics of the enrolled 162 patients: age, sex, body mass index (BMI) at the time of ultrasonography, and prescribed medications were collected as clinical variables. Plasma renin activity (PRA), plasma aldosterone concentration (PAC), estimated glomerular filtration rate (eGFR), and protein level in the urine (U-pro) were collected as laboratory variables. The value of eGFR was calculated based on sex, age, and serum creatinine level with the following equations [12].

$$eGFR[ml/min/\it{1.73m}^{\it{2}}](male) = \it{194} \ast \mathrm{Cr}^{-\it{1.094}} \ast \mathrm{Age}^{-\it{0.287}}$$

$$\it{eGFR}[\it{ml}/\it{min}/\it{1}\it{.73}\,\it{m}^{\it{2}}](\it{female}) = \it{eGFR}\left( \it{male} \right) \ast \it{0}{\it{.739}}$$

SBP and diastolic blood pressure (DBP), both of which were measured by sphygmomanometer, and PP were collected around the time of ultrasonography. Based on these data, ratios including DBP/SBP and PP/SBP were further calculated.

Data from renal color Doppler ultrasonography were acquired from both sides of the kidney and averaged. PSV, EDV, and RI were measured in all of the following four kidney regions: trunk of the renal artery, hilum (renal pedicles), and segmental and interlobar region. PSV was also measured in the aorta (aortic PSV) [13]. RI was calculated from the values of PSV and EDV with the following equation and regarded to represent the peripheral vascular resistance in this study [2].

$${\rm{RI}} \,=\, \frac{{\rm{PSV}} - {\rm{EDV}}}{{\rm{PSV}}}$$

**Fig. 1:**
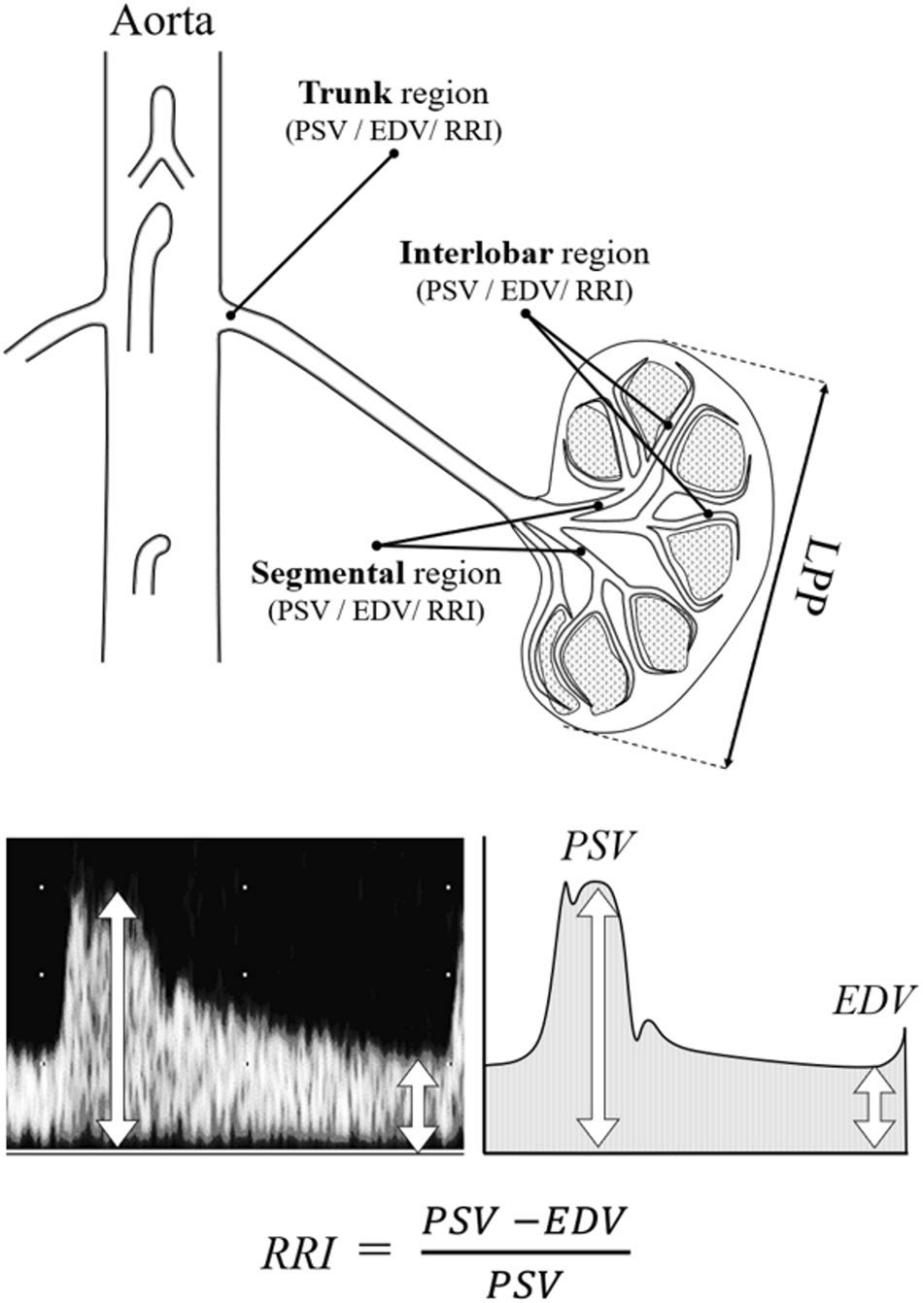
Schema to show the studied four renal regions and the concept of renal resistive index. The studied four regions from the proximal to the distal are as follows: trunk, hilum, segmental, and interlobar. EDV end-diastolic velocity, LPP pole-to-pole kidney length, PSV peak systolic velocity, RRI renal resistive index

The schema to explain the studied renal regions and the components of RI are shown in Fig. 1.

### Compatibility between PP/SBP and DBP/SBP

As shown below, the ratio of PP and SBP is equal to the remainder obtained when the ratio of DBP and SBP (D/S ratio) is subtracted from 1.

$$\frac{\mathrm{PP}}{\mathrm{SBP}} \,=\, \frac{\mathrm{SBP} \,-\, \mathrm{DBP}}{\mathrm{SBP}} \,=\, 1 \,-\, \frac{\mathrm{DBP}}{\mathrm{SBP}}$$

Thus, when we evaluate the correlations between blood pressure and Doppler ultrasonography parameters, the values for PP/SBP and DBP/SBP show the same correlation coefficients as the renal Doppler ultrasonography-related parameters, including RI. With this basis, we employed DBP/SBP (D/S ratio) in this study, rather than PP/SBP, for its computing convenience.

### Institutional review board

All investigative procedures were approved by the Institutional Review Board of Tohoku University Hospital (IRB No. 2015-1-765). The study was performed in compliance with the Helsinki Declaration.

### Statistics and software

Correlation coefficients between blood pressure indices and other clinical and laboratory variables were evaluated with the Spearman’s rank correlation coefficient (*R)*, unless variables showed an apparent nonnormal distribution. The test for no correlation was performed for all studied pairs. Because the test for no correlation was performed simultaneously for more than 50 pairs, *p*-values <0.001were regarded as statistically significant for this study.

Statistical analyses in this study were conducted using SPSS Statistics Base 22 software (IBM, Armonk, NY, USA), JMP Pro 14 (SAS Institute Inc., Cary, NC, USA) and MATLAB R2015a (MathWorks, Natick, MA, USA).

## Results

### Background of the patients

Among the enrolled 162 subjects, 97 were male and 65 were female. Mean ± standard deviation of age and BMI were 55.9 ± 15.7 years and 23.9 ± 3.7 kg/m^2^, respectively. The number of subjects at each CKD stage was as follows: stage 1, 29 subjects; Stage 2, 66 subjects; stage 3A, 24 subjects; stage 3B, 24 subjects; stage 4, 10 subjects; and stage 5, 9 subjects. Among the enrolled subjects, 28 were presently under treatment with oral diuretics, 110 with calcium blockers, and 97 with angiotensin-converting enzyme inhibitor or an angiotensin receptor blocker (ACEI/ARB). Eighty-three of the subjects were with two or more of these medications, whereas 32 subjects were with none of these mediations.

### Correlations between blood pressure indices and other variables

The correlation matrix between the four blood pressure indices (i.e., SBP, DBP, D/S ratio, and PP) and other studied variables is shown in Table 1. The D/S ratio and PP showed significant correlations with age, but the divided components of SBP and DBP did not. U-pro, PRA, PAC, and eGFR did not show significant correlations with the blood pressure-related indices. As to the ultrasonic indices, PSV did not show significant correlations with any of the blood pressure indices. EDV was suggested to show a significant correlation only with the D/S ratio, but the level of correlation was weak. Meanwhile, RI in all four of the studied regions showed significant correlations with the blood pressure indices excluding SBP; however, the D/S ratio showed the strongest correlation with RI.

**Table 1:** Correlation matrix of blood pressure indices and other variables

	SBP	DBP	D/S ratio	PP
Age	0.135 (−0.022 to 0.285)	−0.243 (−0.385 to −0.090)	−0.444** (−0.309 to −0.562)	0.359** (0.214 to 0.488)
BMI	0.145 (−0.016 to 0.299)	0.219 (0.060 to 0.366)	0.105 (−0.057to 0.262)	0.009 (−0.152 to 0.170)
U-pro	0.034 (−0.140 to 0.205)	−0.139 (−0.305 to 0.035)	−0.194 (−0.355 to −0.022)	0.152 (−0.022 to 0.316)
PRA	−0.088 (−0.252 to 0.082)	−0.042 (−0.209 to 0.128)	0.030 (−0.140 to 0.197)	−0.079 (−0.244 to 0.091)
PAC	−0.018 (−0.187 to 0.151)	0.064 (−0.106 to 0.230)	0.090 (−0.081 to 0.255)	−0.070 (−0.236 to 0.101)
eGFR	−0.151 (−0.301 to 0.007)	0.023 (−0.135 to 0.180)	0.184 (0.027 to 0.332)	−0.206 (−0.352 to −0.050)
Trunk				
PSV	−0.020 (−0.180 to 0.140)	−0.203 (−0.351 to –0.045)	−0.221 (−0.367 to –0.063)	0.133 (−0.027 to 0.287)
EDV	−0.102 (−0.257 to 0.059)	0.228 (0.071 to 0.374)	0.371** (0.225 to 0.501)	−0.304* (−0.443 to −0.152)
RI	0.101 (−0.061 to 0.258)	−0.412** (−0.537 to −0.269)	−0.585** (−0.681 to −0.468)	0.444** (0.305 to 0.565)
Hilum				
PSV	−0.020 (−0.184 to 0.145)	−0.090 (−0.250 to 0.075)	−0.092 (−0.252 to 0.074)	0.044 (−0.121 to 0.207)
EDV	0.045 (−0.120 to 0.208)	0.260 (0.100 to 0.407)	0.235 (0.074 to 0.384)	−0.144 (−0.301 to 0.020)
RI	0.084 (−0.082 to 0.244)	−0.385** (−0.517 to −0.236)	−0.542** (−0.649 to −0.415)	0.399** (0.252 to 0.529)
Segmental				
PSV	0.061 (−0.100 to 0.218)	−0.123 (−0.277 to 0.038)	−0.218 (–0.365 to −0.060)	0.181 (0.021 to 0.331)
EDV	0.014 (−0.171 to 0.174)	0.228 (0.071 to 0.375)	0.253 (0.096 to 0.397)	−0.172 (−0.324 to −0.012)
RI	0.126 (−0.034 to 0.279)	−0.413** (–0.537 to −0.272)	−0.641** (−0.726 to −0.536)	0.513** (0.385 to 0.621)
Interlobar				
PSV	0.065 (−0.096 to 0.222)	−0.084 (−0.241 to 0.076)	−0.178 (−0.329 to −0.019)	0.146 (−0.014 to 0.299)
EDV	−0.011 (−0.171 to 0.150)	0.302* (0.149 to 0.441)	0.364** (0.216 to 0.495)	−0.250 (–0.394 to −0.093)
RI	0.116 (−0.045 to 0.270)	−0.402** (–0.528 to −0.259)	−0.612** (−0.703 to −0.502)	0.461** (0.325 to 0.578)
LPP	0.034 (−0.124 to 0.191)	0.194 (0.037 to 0.341)	0.189 (0.032 to 0.336)	−0.110 (−0.263 to 0.049)
Aortic PSV	−0.000 (−0.158 to 0.157)	−0.064 (−0.219 to 0.095)	−0.078 (−0.233 to 0.080)	0.050 (−0.109 to 0.206)

In all four of the regions where the renal Doppler ultrasonography was performed, RI showed strong negative correlations with D/S ratio and moderate correlations with DBP and PP

*BMI* body mass index, *D/S ratio* ratio of diastolic to systolic blood pressure, *DBP* diastolic office blood pressure, *EDV* end-diastolic velocity, *eGFR* estimated glomerular filtration rate, *LPP* pole-to-pole kidney length, *PAC* plasma aldosterone concentration, *PP* pulse pressure, *PRA* plasma renin activity, *PSV* peak systolic velocity, *RI* resistive index, *SBP* systolic office blood pressure, *U-pro* protein level in urine

*p < 0.001; **p < 0.0001

### Partial correlation coefficients after setting age as covariate

Because a significant correlation between age and D/S ratio was suggested, partial correlation coefficients between ultrasonic indices and blood pressure indices were comprehensively evaluated after setting age as a covariate. The acquired partial correlation matrix is shown in Table 2. Moderate levels of negative correlations were confirmed between the RI in all four of the kidney regions and D/S ratio.

**Table 2:** Partial correlation matrix by setting age as the covariate

	SBP	DBP	D/S ratio	PP
Trunk				
PSV	−0.0174 (*p* = 0.8325)	−0.215 (*p* = 0.0082)	−0.256 (*p* = 0.0016)	0.151 (*p* = 0.0654)
EDV	−0.050 (*p* = 0.5450)	0.138 (*p* = 0.0928)	0.220 (*p* = 0.0069)	−0.177 (*p* = 0.0305)
RI	0.054 (*p* = 0.5139)	−0.342** (*p* < 0.0001)	−0.481** (*p* < 0.0001)	0.343** (*p* < 0.0001)
Hilum				
PSV	0.022 (*p* = 0.7958)	−0.172 (*p* = 0.0406)	−0.254 (*p* = 0.0023)	0.165 (*p* = 0.0493)
EDV	0.117 (*p* = 0.1645)	0.183 (*p* = 0.0295)	0.060 (*p* = 0.4752)	0.008 (*p* = 0.929)
RI	0.024 (*p* = 0.7812)	−0.324** (*p* < 0.0001)	−0.430** (*p* < 0.0001)	0.288* (*p* = 0.0005)
Segmental				
PSV	0.066 (*p* = 0.424)	−0.136 (*p* = 0.0981)	−0.267 (*p* = 0.0010)	0.212 (*p* = 0.0091)
EDV	0.067 (*p* = 0.418)	0.156 (*p* = 0.0581)	0.115 (*p* = 0.1621)	−0.043 (*p* = 0.5996)
RI	0.036 (*p* = 0.665)	−0.336** (*p* < 0.0001)	−0.496** (*p* < 0.0001)	0.354** (*p* < 0.0001)
Interlobar				
PSV	0.062 (*p* = 0.4480)	−0.081 (*p* = 0.3226)	−0.188 (*p* = 0.0213)	0.147 (*p* = 0.0720)
EDV	0.054 (*p* = 0.5108)	0.209 (*p* = 0.0106)	0.191 (*p* = 0.0200)	−0.097 (*p* = 0.2381)
RI	0.043 (*p* = 0.5977)	−0.331** (*p* < 0.0001)	−0.483** (*p* < 0.0001)	0.329** (*p* < 0.0001)

*D/S ratio* ratio of diastolic to systolic blood pressure, *EDV* end-diastolic velocity, *PP* pulse pressure, *PSV* peak systolic velocity, *RI* resistive index

*p < 0.001; **p < 0.0001

### Scatter plots of RI and blood pressure indices

**Fig. 2:**
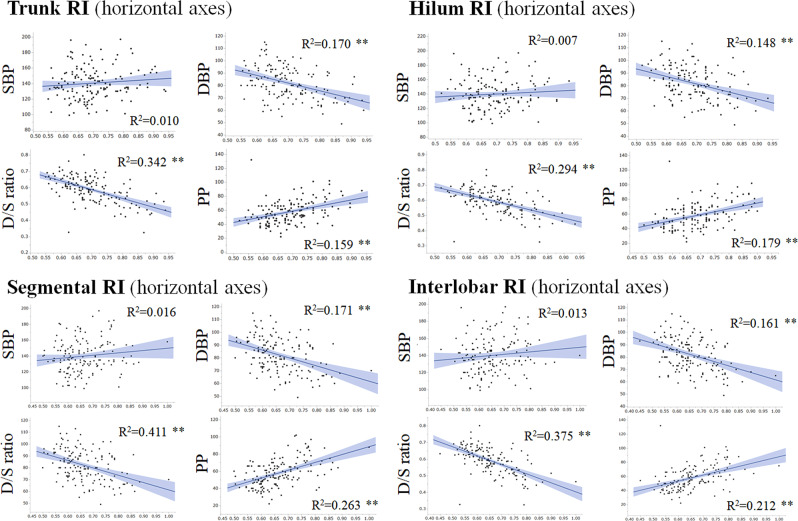
Scatter plots of RI in each region and blood pressure parameters. To visually confirm the correlations between peripheral vascular resistance—represented by RI in this study—and blood pressure indices, scatter plots between each pair of these variables were generated. Shaded gray color ranges show the 95% confidence interval of the regression lines. *R*^2^ is the coefficient of determination, which stands for the percentage of variability in the outcome data that can be explained by the model fit. DBP diastolic blood pressure, D/S ratio ratio of diastolic to diastolic blood pressure, PP pulse pressure, RI resistive index, SBP systolic blood pressure

Scatter plots for RI in each of the four kidney regions and the examined blood pressure indices are shown in Fig. 2. Among the four blood pressure indices, the D/S ratio showed the strongest correlations with RI values in the four kidney regions. DBP and PP also showed significant correlations with RI, but they were only weak to moderate correlations; meanwhile, SBP did not show any correlation with RI.

### Pulse pressure and mean arterial pressure

Because there was a significant correlation between the PP value and D/S ratio in the enrolled subjects (Pearson’s R = −0.864, *p* < 0.0001), we also evaluated the partial correlation coefficients by using PP, D/S ratio, and the averaged RI value of the four regions. We found that the partial correlation coefficient between PP and the averaged RI value was not statistically significant (R = −0.112, *p* = 0.198), whereas the partial correlation coefficient between D/S ratio and the averaged RI value was significant (R = −0.474, *p* < 0.0001).

In addition, we evaluated the partial correlation coefficients by using mean arterial pressure (MAP), D/S ratio, and the averaged RI value of the four regions. Our results indicated that the partial correlation coefficient between MAP and the averaged RI value was very weak (R = −0.215, *p* = 0.012), whereas the partial correlation coefficient between D/S ratio and the averaged RI value was much stronger (*R* = −0.649, *p* < 0.0001).

### Correlation between RI and D/S ratio among subjects without CKD

**Fig. 3:**
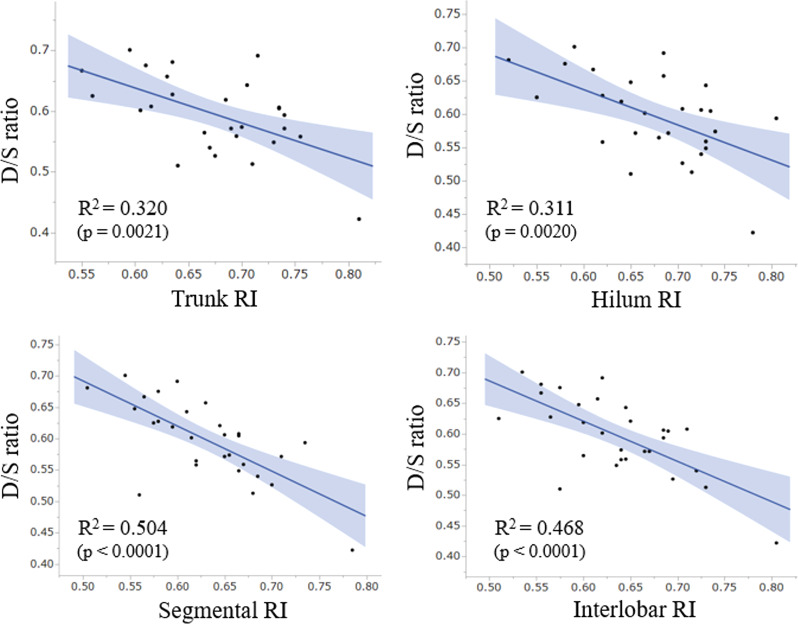
Scatter plots between RI of four kidney regions and D/S ratio within subjects without renal dysfunction (CKD stage 1). The negative correlation between peripheral vascular resistance and the D/S ratio was confirmed among CKD patients without renal dysfunction (CKD stage 1), suggesting that this negative correlation could be generalized to the patient population without advanced CKD. The shaded gray-color ranges show the 95% confidence interval of the regression lines. *R*^2^ is the coefficient of determination. *D/S ratio* ratio of diastolic to systolic blood pressure, *RI* resistive index

To confirm that the relationship between renal vascular resistance and the D/S ratio could be generalized to the population without advanced CKD, we also checked the correlation in 29 patients without renal dysfunction (i.e., CKD stage 1). Statistically significant negative correlations were confirmed in all four of the studied kidney regions. The calculated correlation coefficients with D/S ratio were −0.57 for trunk RI (*p* = 0.002), −0.56 for hilum RI (*p* = 0.002), −0.71 for segmental RI (*p* < 0.0001), and −0.68 for interlobar RI (*p* < 0.0001). The scatter plots of these measurements in each kidney region are shown in Fig. 3. RI in the segmental and interlobar regions showed the strongest correlations with D/S ratio. These findings showed that the negative correlation between renal vascular resistance and the D/S ratio could be applied to the patient population without kidney dysfunction.

For reference, to compare the practicality of D/S ratio and of PP as a marker of renal vascular resistance, we also evaluated the correlation between PP and RI in the 29 subjects without renal dysfunction. The calculated correlation coefficients with PP were 0.35 for trunk RI (*p* = 0.082), 0.34 for hilum RI (*p* = 0.082), 0.57 for segmental RI (*p* = 0.002), and 0.50 for interlobar IR (*p* = 0.008). These coefficients of determination were much weaker than those with D/S ratio in all four of the studied regions, suggesting that D/S ratio would be a better marker of renal vascular resistance than PP.

### Correlation between RI and D/S ratio among subjects with CKD

In addition, to confirm whether the strong negative correlation between D/S ratio and RI is irrespective of the existence of CKD, we evaluated the correlations in the 67 patients with CKD stages 3A or higher. Our results found that the calculated correlation coefficients with D/S ratio were −0.74 for trunk RI, −0.66 for hilum RI, −0.61 for segmental RI, and −0.65 for interlobar IR (*p* < 0.0001 for all four regions). These correlations were as high as those in the subjects without CKD, suggesting that the strong negative correlation between D/S ratio and renal RI stands irrespective of renal function.

### Correlation between D/S ratio and RI by the prescribed medications

To exclude the possibility that medication interfered with the results, correlation coefficients between D/S ratio and RI were studied by the prescribed medications. In those with oral diuretics (*n* = 28), −0.52 for trunk RI (*p* = 0.006), −0.33 for hilum RI (*p* = 0.13), −0.72 for segmental RI (*p* < 0.0001), and −0.40 for interlobar RI (*p* = 0.042). In those with calcium-blockers (*n* = 110), −0.59 for trunk RI, −0.57 for hilum RI, −0.64 for segmental RI, and −0.61 for interlobar RI (*p* < 0.0001 for all four regions). In those with ACEI/ARB (*n* = 97), −0.59 for trunk RI, −0.55 for hilum RI, −0.65 for segmental RI, and −0.60 for interlobar RI (*p* < 0.0001 for all four regions). In those with no medications (*n* = 32), −0.48 for trunk RI (*p* = 0.012), −0.39 for hilum RI (*p* = 0.041), −0.62 for segmental RI (*p* = 0.0003), and −0.61 for interlobar RI (*p* = 0.0006). Our results concluded that the presently prescribed medications did not interfere with the acquired results of this study.

## Discussion

In this study, we showed that the D/S ratio value, rather than PP or MAP, is the most appropriate index that can be used as a marker for peripheral vascular resistance, indicated by renal RI in this study. Up to now, PP and MAP have been suggested to correlate with renal RI [4, 10, 11, 14], but D/S ratio has never been evaluated as a marker of vascular resistance or for the possible association with macrovascular diseases. Based on the results of this study, though D/S ratio certainly shows strong correlations with these two indices, D/S ratio would be a better index than the other two indices to reflect renal RI. Another conclusion of this study was that DBP represents the vascular resistance to some extent, but SBP does not at all. SBP seems to be widely regulated by cardiac output and proximal arterial capacity [15, 16], whereas DBP is mainly regulated by peripheral vascular capacity and resistance based on Poiseuille’s law as PP would be produced by the draining step of arterial blood into the periphery [17–20]. This supports the conclusion that DBP would represent the peripheral vascular resistance much more than SBP.

Another notable finding of this study was that, among the three ultrasonic indices (i.e., EDV, PSV, and RI), only the RI value showed significant correlations with any of the studied blood pressure indices. Moreover, the achieved dataset suggested that renal function would be better reflected by EDV than by RI, which will be reported in detail in a future publication. These facts suggest that the most important ultrasonic index in the management of CKD would be EDV, but in a follow-up for hypertension RI would be more important. Conversely, the D/S ratio would be the most important blood pressure index among the studied four indices to estimate the degree of intrarenal vascular resistance. Because vascular conductivity can be represented by the inverse of vascular resistivity, the S/D ratio can be regarded as a marker of vascular flexibility.

$$	\mathrm{Vascular}\;\mathrm{conductivity} \\ 	\quad= {\frac{1}{{\mathrm{Vascular}}\;\mathrm{resistivity}}} \propto {\frac{1}{{(\mathrm{DBP/SBP})}}} \,=\, {\frac{{\mathrm{SBP}}}{{\mathrm{DBP}}}}$$

Hence, EDV should be used in the follow-up in management of CKDs, whereas RI, DBP, and the D/S ratio should be monitored and used in follow-ups in hypertensive patients. Because measurements of blood pressure at a single time are vulnerable to variation, the average of the D/S ratio based on multiple measurements in a specific time period should be first achieved to acquire reliable data.

There are some limitations to this study. First, the measurement of vascular resistance was only performed in the renal artery and kidney. If we try to generalize the results of this study to systemic vascular resistance (SVR), we need to employ color Doppler ultrasonography in other organs and arteries across the body. For example, obtaining splenic RI values together with renal RI would be practical to differentiate extra from intrarenal influences to the value of renal RI. Simply checking the ankle brachial index or pulse wave velocity could also be helpful to estimate the SVR. Theoretically, the following equations to estimate SVR, proposed in some previous reports, could also be useful [10].

$$	\mathrm{SVR} \, = \left( {\frac{\mathrm{MAP} - \mathrm{CVP}}{\mathrm{CO}}} \right) \,\times\, 80\\ 	( \mathrm{CO},{\mathrm{cardiac}\;\mathrm{output}};{\mathrm{CVP}},{\mathrm{central}\;\mathrm{venous}\;\mathrm{pressure}}; \\ 	\quad{\mathrm{MAP}},\;{\mathrm{mean}\;\mathrm{arterial}\;\mathrm{pressure}} )$$

Another limitation is that the clinical significance of renal vascular resistance or SVR is not entirely clear at present. Although higher peripheral vascular resistance surely seems to result in negative outcomes in systemic organs and tissues, whether peripheral vascular resistance is strongly associated with each specific disease (e.g., hypertension, ischemic heart diseases, CKD, and chronic heart diseases) is still inconclusive [11, 21].

In conclusion, D/S ratio was shown to represent the degree of renal vascular resistance, irrelevant to the presence of renal dysfunction or the type of prescribed medications, whereas PP or MAP did not. In long-term follow-ups of hypertensive patients, D/S ratio should be evaluated to estimate the degree of renal vascular resistance irrespective of renal function.

### Summary

#### What is known about the topic

- Increased intrarenal vascular resistance, which can be estimated by intrarenal color Doppler ultrasonography, is known to accompany the progression of chronic kidney diseases (CKD) and hypertension.
- Pulse pressure (PP) and mean arterial pressure (MAP) have been suggested to correlate with renal resistive index (RI) in some previous reports.

#### What this study adds

- Ratio of diastolic to systolic blood pressure (D/S ratio) more strongly correlates with renal RI than PP or MAP.
- A strong negative correlation between D/S ratio and renal RI was confirmed, no matter of the measured renal region, presence of renal dysfunction, or the prescribed medications.
